# Functional Divergence among Silkworm Antimicrobial Peptide Paralogs by the Activities of Recombinant Proteins and the Induced Expression Profiles

**DOI:** 10.1371/journal.pone.0018109

**Published:** 2011-03-29

**Authors:** Wanying Yang, Tingcai Cheng, Mingqiang Ye, Xiaojuan Deng, Huiyu Yi, Yadong Huang, Xiang Tan, Dong Han, Bo Wang, Zhonghuai Xiang, Yang Cao, Qingyou Xia

**Affiliations:** 1 Laboratory of Insect Molecular Biology and Biotechnology, Guangdong Provincial Key Laboratory of Agro-animal Genomics and Molecular Breeding, College of Animal Science, South China Agricultural University, Guangzhou, China; 2 Institute of Agriculture and Life Science, Chongqing University, Chongqing, China; 3 Institute of Sericulture and Systems Biology, Southwest University, Chongqing, China; 4 Biopharmaceutical Research and Development Center, Jinan University, Guangzhou, China; 5 The Sericulture & Agri-Food Research Institute, Guangdong Academy of Agricultural Sciences, Guangzhou, China; Louisiana State University, United States of America

## Abstract

Antimicrobial peptides are small-molecule proteins that are usually encoded by multiple-gene families. They play crucial roles in the innate immune response, but reports on the functional divergence of antimicrobial peptide gene families are rare. In this study, 14 paralogs of antimicrobial peptides belonging to cecropin, moricin and gloverin families were recombinantly expressed in pET expression systems. By antimicrobial activity tests, peptides representing paralogs in the same family of cecropin and moricin families, displayed remarkable differences against 10 tested bacteria. The evolutionary rates were relatively fast in the two families, which presented obvious functional divergence among paralogs of each family. Four peptides of gloverin family had similar antimicrobial spectrum and activity against tested bacteria. The gloverin family showed similar antimicrobial function and slow evolutionary rates. By induced transcriptional activity, genes encoding active antimicrobial peptides were upregulated at obviously different levels when silkworm pupae were infected by three types of microbes. Association analysis of antimicrobial activities and induced transcriptional activities indicated that the antimicrobial activities might be positively correlated with induced transcriptional activities in the cecropin and moricin families. These results suggest that representative *BmcecB6*, *BmcecD* and *Bmmor* as the major effector genes have broad antimicrobial spectrum, strong antimicrobial activity and high microbe-induced expression among each family and maybe play crucial roles in eliminating microbial infection.

## Introduction

In insects, innate immunity is the first line of defense against invading microbes, and antimicrobial peptides (AMPs) play crucial roles in killing invaders and preventing infection. Usually they have a low molecular weight, high heat stability, a broad spectrum, and high antimicrobial activity. Most AMPs are encoded by multiple gene families, as seen in the completed genomic sequences of several insects, such as the *cecropin* and *drosomycin* families in *Drosophila melanogaster* [1], the *cecropin*, *moricin* and *gloverin* families in *Bombyx mori* [2], [3]. The duplication of AMP genes frequently occurs through unequal crossing-over events [4]. The multitudinous AMPs probably maximize the host defensive capability against microbes.

Seven kinds of AMPs have been identified in *Drosophila* [5]. Most of the genes show inducible expression regulated by the Toll and Imd signaling pathways [6]. However, the peptides have remarkably different biological functions. Generally, cecropins have strong antimicrobial activity against gram-positive and gram-negative bacteria [7], and weak activity against fungi of cecropinA [8], while drosomycin exhibits potent activity against filamentous fungi [9]. Drosocin and attacins have direct activity against gram-negative bacteria [10], [11], defensin against gram-positive bacteria [12], diptericin against both gram-positive and gram-negative bacteria [13], and metchnikowin against gram-positive bacteria and filamentous fungi [14]. The different peptide classes vary in their mechanisms of microbial recognition and killing, but all peptides interact directly with microbes, resulting in the potential coordinate evolution of host genes with microbes. Moreover, in previous studies, evidence for adaptive evolution among *Drosophila* AMPs has been found by molecular population and comparative genomic analysis [1], [15]. AMPs have extensive gene duplication and rapid gene turnover, but positive selected sites are absent [4]. Both rapid gene duplication and positive selection have been found in AMPs from frogs [16], [17].

Species-specific rapid changes in AMP gene copy number are found in several insect gene families [18]. However, functional divergence among paralogs in the same gene family has rarely been reported with experimental support. The *drosomycin* (*Drs*) multigene family contains seven paralogs in *D. melanogaster*, a well model for gene evolution of insect AMPs [4]. In previous studies, *Drs* was strongly up-regulated by infection with fungi and gram-positive bacteria, and weakly up-regulated by gram-negative bacteria [19], [20], while *Dro5* was also significantly up-regulated by infection of fungi or mixed bacteria [21]. Temporal-spatial expression analysis showed that *Dro2* and *Drs* were expressed in larvae, pupae and adult, and *Dro3*, *Dro4* and *Dro5* were expressed in larvae and adult, while transcripts of *Dro1* and *Dro6* were not detected in any developmental stages [22].

In previous study, by testing the antimicrobial activity of recombinant proteins and induced expression profile in *Drosophila* adults, we found the functional divergence of *drosomycin* family and explored the coevolutionary mechanism in insect immune defense. The results showed seven paralogs of *drosomycin* family revealed obviously different antifungal activity against seven fungal strains [23], and distinct induced expression profile by four different microbes or septic injury [24]. *Drs* has the strongest antifungal activity against the seven tested fungi, and the highest basic and induced expression. The other paralogs have obviously different antifungal activity. These data indicate a possible relationship among *drosomycin* paralogs. A member has a broader antifungal spectrum and also has higher constitutive and induced expression activity. The characteristics on the *drosomycin* family raised the question how synergic resistance against microbes occurs inside AMP family.

In the silkworm genome, several genes encode AMPs, including *cecropin*, *moricin*, *gloverin*, *attacin*, *lebocin* and *defensin* [2], [3]. *Cecropin*, *moricin* and *gloverin* are typical multigene families comprised of 11, 12 and 4 genes, respectively. The *cecropin* family is classified into five subtypes (A–E). Six B-subtype *cecropins* are clustered on chromosome 26, suggesting that the expansion of B-subtype genes occurred by gene duplication [3]. The *moricin* family is composed of one *Bmmor* [25], [26], [27], and three *moricin*-like A and eight *moricin*-like B genes [2], [3]. The similarity of mature proteins is very low among moricin subtypes at the amino acid level. Four genes encode gloverin antimicrobial proteins that are reported to have weak but significant antimicrobial activities against *Escherichia coli* [28], Three genes (*Bmglv2–4*) derived from *Bmglv1* by three gene duplication events are expressed in all embryonic stages, but the ancestral *Bmglv1* gene is not, suggesting that the derived genes have gained embryonic expression and novel function [29].

In this study, the two principal questions were: (i) whether both antimicrobial and induced expression activities were different among AMP families' paralogs; and (ii) whether the relationship between antimicrobial and induced expression activities found in the *drosomycin* family was common to insect AMP families. Using the silkworm as a model [30], [31], this is the first report of a systematic investigation of the antimicrobial and induced expression activities among paralogs of multigene families encoding the cecropin, moricin and gloverin AMPs. By *in vitro* testing the antimicrobial activity of recombinant AMPs and by Real-time RT-PCR testing induced expression profiles of these AMP genes after infection of day-2 pupae with three types of microbes, our results indicated that these paralogs among either *cecropin* family or *moricin* family have obviously diversified in antimicrobial function and show a correlation between broad antimicrobial spectrum and strong antibacterial activity with high induced expression level of AMP genes, similar to *drosomycin* family in *Drosophila*. Four paralogs of gloverin family have very similar antimicrobial activities but distinctly induced expression profiles against different microbe. Consequently, whether is the correlation common between antimicrobial activity and induced transcriptional level of AMPs families? More evidence from AMP families is needed to further determine the functional divergence in insect immune defense.

## Results

### Function divergence of antimicrobial activities of AMP families

**Figure 1:**
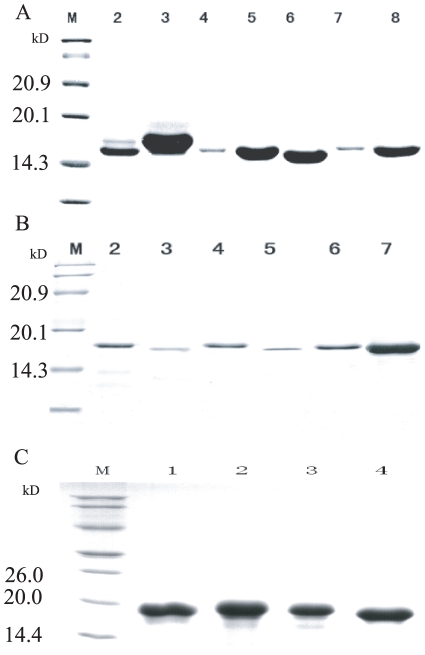
SDS-PAGE analysis of the fusion protein of Cecropin (A), Moricin (B) and Gloverin (C) family purified by Sephadex G-10 Sepharose chromatogramphy. A) Cecropin family: M: Protein molecular weight marker; 2–8: The purified fusion protein BmcecA1, BmcecC, BmcecE, BmcecD1, BmcecD2, BmcecB6 and CecropinD by Sephadex G-10 Sepharose chromatogramphy. B) Moricin family: M: Protein molecular weight marker; 2–8: The purified fusion protein Bmmor, BmmorLA1, Msmor, BmmorLB5, BmmorLB6 and BmmorLB1 by Sephadex G-10 Sepharose chromatogramphy. C) Gloverin family: M: Protein molecular weight marker; 1–4: The purified fusion protein Bmglv1, Bmglv2, Bmglv3 and Bmglv4 by Sephadex G-10 Sepharose chromatogramphy. The concentration of separating gel was 15%.

**Figure 2:**
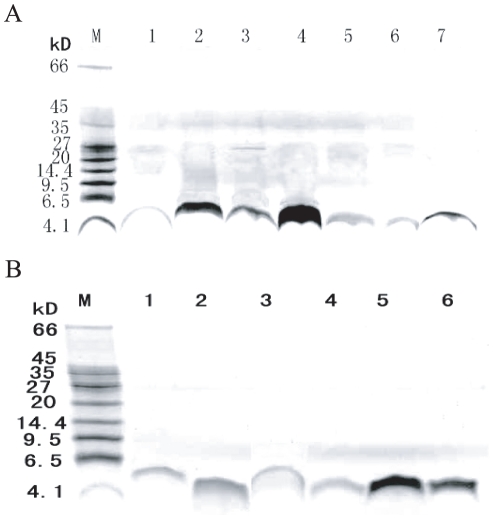
The cecropins (A) and moricins (B) digested by enterokinase and purified twice molecular ultrafiltrations. These purified proteins were tested by Tricine-SDS-PAGE (16.5% separating gel). A) The molecular weights of cecropins were 4.0 kD. M: Protein molecular weight marker; 1–7: The purified BmcecA1, BmcecC, BmcecE, BmcecD1, BmcecD2, BmcecB6 and CecropinD. B) The molecular weights of moricins were about 4.7 kD. M: Protein molecular weight marker; 1–7: The purified fusion protein Bmmor, BmmorLA1, Msmor, BmmorLB5, BmmorLB6 and BmmorLB1.

The mature peptides were very similar among each gene family (*cecropin*, *moricin* and *gloverin* family) in the silkworm genome. We chose representative paralogous genes to express recombinant proteins from three families, *viz.* five *cecropin* paralogs (*Bmcec*A1, B6, C, D, E), five *moricin* paralogs (*Bmmor*, LA1, B1, B5, B6), and four *gloverin* paralogs (*Bmglv*1–4). These recombinant proteins were mainly expressed with intercellular soluble form in *E. coli* Rosetta™(DE3) (Fig. S1). The products of cecropins and moricins contained the fragment of Trx(105aa)-His·Tag (6aa)-Thrombin-S·Tag in the N terminal, whereas the products of gloverins contained the 8aa fragment of Leu-Glu-6His·Tag in the C terminal. So, the recombinant proteins were purified by Ni-NTA affinity chromatography (Fig. S2) and sephadex G-10 chromatography desalination for *cecropin* (Fig. 1A), *moricin* (Fig. 1B) and *gloverin* families (Fig. 1C). Then, the products of cecropins and moricins were digested by enterokinase (EK) and remained two extra residues (Ala-Met) in their N terminals. They were purified by twice molecular ultrafiltrations (molecular weight cut-off 10.0 kD and 3.0 kD). The molecular weights of purified cecropins (Fig. 2A) and moricins (Fig. 2B) were 4.0 kD and 4.7 kD, respectively. Tobacco hornworm Moricin (*Manduca sexta*) AMP (Msmor) was expressed as a control to facilitate comparison of the antimicrobial characteristics of the *moricin* family.

**Figure 3:**
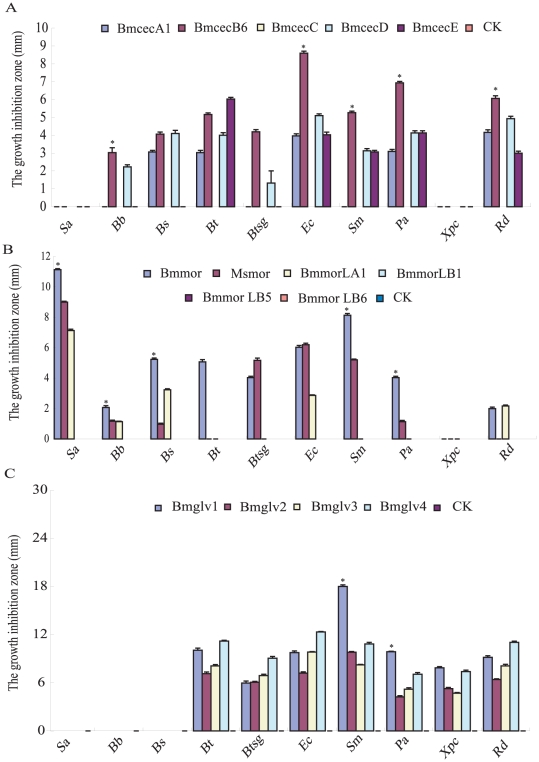
The antimicrobial spectrum of paralogs among each AMP gene family. The following microbes were used to test antimicrobial activities: Gram-positive bacteria: *S. aureus (Sa)*, *B. bombysepticus (Bb)*, *B. subtilis (Bs)*, *B. thuringiensis (Bt)*, *B. thuringiensis subsp. galleriae (Btsg)*; Gram-negative bacteria: *E. coli (Ec)*, *S. marcescens (Sm)*, *P. aeruginosa (Pa)*, *X. pv. campestris (Xpc)*, *R. dolaanacearum (Rd)*. Each sample (5 µmol/L, 10 µL) was dropped into the 2.7 mm pore on LB-medium plates and incubated at 37°C for 24 h as shown in Figure S3 for moricin family. The measure unit is millimeter (mm). The ring diameter indicated that the average size of the clear area around the bacteria (n = 3). Differences were significant at *P<0.01 among each recombinant AMP family.

By ultrasensitive radial diffusion (Fig. S3) and minimum inhibitory concentrations (MICs), the antimicrobial spectrum and biological activity of above recombinant antimicrobial proteins were tested against five Gram-positive bacteria and five Gram-negative bacteria. The results indicated three antimicrobial peptide families had different antimicrobial spectrum. Cecropin family did not have activity against *Staphylococcus aureus* and *Xanthomons pv. campestris*, moricin family did not have activity against *X. pv. campestris*, and gloverin family did not have activity against *S. aureus*, *Bacillus bombysepticus* and *Bacillus subtilis* (Fig. 3). Moreover, five recombinant AMPs of cecropin family had clearly different characteristics in antimicrobial spectrum and activity, as well as the moricin family. Four recombinant AMPs of gloverin family had similar characteristics (Fig. 3 and Table 1).

**Table 1:** Minimal growth inhibition concentrations (MICs) of antimicrobial peptides against the ten tested microbes.

	Gram-positive bacteria					Gram-negative bacteria				
	*S. aureus*	*B. bombysepticus*	*B. subtilis*	*B. thuringiensis*	*B. thuringiensis subsp. galleriae*	*E. coli*	*S. marcescens*	*P. aeruginosa*	*X. campestris*	*R. dolaanacearum*
BmcecA1	-	-	2.5	2.5	-	2.5	-	2.5	-	2.5
BmcecB6	-	2.5	2.5	1.25	1.25	0.625	0.625	0.625	-	1.25
BmcecD	-	2.5	2.5	1.25	2.5	1.25	2.5	1.25	-	1.25
BmcecE	-	-	-	1.25	-	2.5	2.5	2.5	-	2.5
Bmmor	0.625	1.25	0.625	1.25	1.25	1.25	0.625	1.25	-	1.25
BmmorLA1	1.25	2.5	2.5	-	-	2.5	-	-	-	1.25
Msmor	1.25	2.5	2.5	-	1.25	2.5	1.25	2.5	-	-
Bmglv1	-	-	-	1.4	1.6	1.4	1.2	1.4	1.6	1.4
Bmglv2	-	-	-	1.6	1.6	1.6	1.6	1.8	1.8	1.6
Bmglv3	-	-	-	1.6	1.6	1.4	1.6	1.8	1.8	1.6
Bmglv4	-	-	-	1.4	1.6	1.4	1.4	1.6	1.6	1.4

Among five recombinant cecropins, BmcecB6 representing B subtype had wide antimicrobial spectrum and the strongest biological activity against eight of ten tested bacteria. Only two bacteria (*S. aureus* and *X. pv. campestris*) were not inhibited by BmcecB6 (Fig. 3). BmcecD had a similar antimicrobial spectrum as BmcecB6. BmcecA1 and BmcecE had a similar antimicrobial spectrum against five of ten tested bacteria, whereas, BmcecC had no ability to inhibit the growth of these tested bacteria. Interestingly, the antimicrobial activity of BmcecB6 was remarkably higher than those of other four paralogs by ultrasensitive radial diffusion assays (*p*<0.01) (Fig. 3A). By MICs assays, BmcecB6 and BmcecD had stronger antimicrobial activities than BmcecA1 and BmcecE, moreover, the antimicrobial activities against four bacteria (*Bacillus thuringiensis subsp. Galleriae*, *E. coli*, *Serratia marcescens* and *Pseudomonas aeruginosa*) of BmcecB6 were relatively stronger than those of BmcecD (Table 1). Overall, BmcecB6 and BmcecD showed relatively strong antimicrobial activities among cecropin members.

Moricin is a lepidoptera-specific AMP with wide antimicrobial characteristics. The antimicrobial spectrum and activity were obviously different among *B. mori* moricins and *M. sexta* moricin [32]. Bmmor had strongly antimicrobial activity against nine of ten tested bacteria, except for *X. pv. campestris* (Fig. 3B). Msmor had antimicrobial activity against seven of ten tested bacteria, but no activity against *Bacillus thuringiensis*, *X. pv. campestris* and *Ralstonia dolaanacearum* (Fig. 3). Moreover, Bmmor had remarkably stronger activity than that of Msmor in MICs results (Table 1). Among the silkworm moricin family, the antimicrobial spectrum of BmmorLA1 was narrower than that of Bmmor, only against three tested bacteria (Fig. 3B), and the antimicrobial activity of BmmorLA1 was remarkably lower than that of Bmmor (*p*<0.01) (Fig. 3B, Table 1). The moricin B subtype had very low similarity with the A subtype at the amino acid sequence level. Three paralogs of moricin B subtype seemed inactive against these tested bacteria (Fig. 3B). Surmisably, Bmmor showed the characteristics of the strongest antimicrobial activity among moricin family.

Gloverins are glycine-rich antimicrobial proteins, reported from lepidopteran insects [33], [34]. Previous studies showed gloverins only had antimicrobial activity against Gram-negative bacteria [35], [36]. The silkworm genome contains four intact *gloverin* genes (*Bmglv1–4*) and three incomplete genes lacking C-terminal or N-terminal portions. Interestingly, four recombinant proteins (Bmglv1–4) had completely similar antimicrobial spectra against five Gram-negative bacteria (Fig. 3C). Also, they had antimicrobial activities against two Gram-positive bacteria, *B. thuringiensis* and *B. thuringiensis subsp. Galleriae*. In particular, the diameter of the clear area reached 18 mm when Bmglv1 was incubated with *S. marcescens* at 37°C for 24 h (Fig. 3). A previous report found that Bmglv1 had a stronger antimicrobial activity than Bmglv2 by incubation with *E. coli* [28]. However, their antimicrobial activities were very similar by MICs assay (Table 1).

By comparative analysis of antimicrobial assays, we found that BmcecB6 and BmcecD of cecropin family and Bmmor of moricin family had relatively wider antimicrobial spectrum and/or stronger antimicrobial activity than other paralogs of each family, suggesting that they were likely to be major genes with main functions in the antimicrobial defenses in the two gene families, while other members of each family might function as facilitators of antimicrobial defense.

### Induced expression patterns of AMP gene families

**Figure 4:**
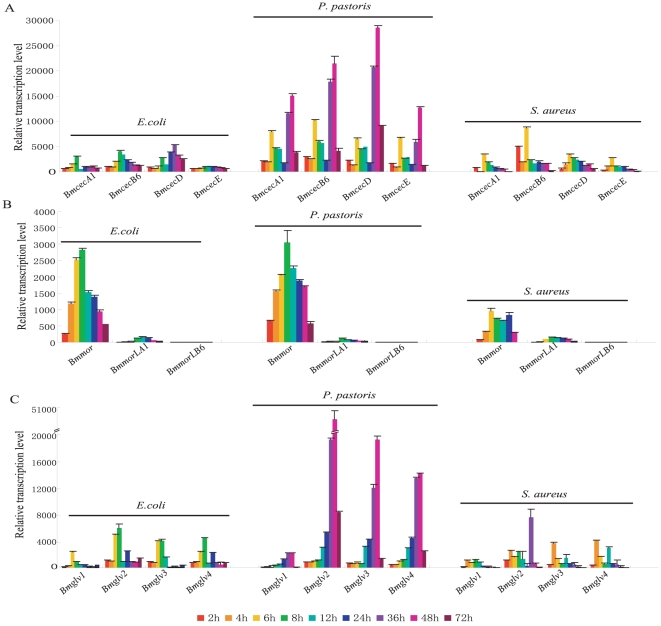
The transcription activity of AMP gene families in *B. mori* induced by various microbes. Total RNAs were isolated using TRIzol Reagent (Invitrogen) from the day-2 pupae collected at 2, 4, 6, 8, 12, 24, 36, 48 and 72 h after injection with *E. coli*, *S. aureus* and *P. Pastoris*, respectively. Quantitative real-time RT-PCR was performed for the detection of the expression levels of these genes in silkworm pupae with SYBR Green. The expression levels were normalized to *actin3* in the samples. The experiments were done in triplicate and the error bars represented standard deviation.

To investigate whether induced expression patterns were different among the 12 genes, we performed quantitative real-time PCR to analyze their expression patterns in pupae infected with the Gram-negative bacterium *E. coli*, the Gram-positive bacterium *S. aureus*, and the fungus *Pichia pastoris*. When pupae were infected, most genes were strongly induced, with obvious differences in degree (Fig. 4). In the three gene families, induced expression by the fungus was substantially higher than induction by the bacteria. Especially *cecropin* and *gloverin* families, the induced peak values after fungus infection were 5–10 times higher than bacteria (Fig. 4A and 4C). In temporal patterns, expression quantities peaked from 6 to 8 h after bacterial infection, and tended to decrease later (Fig. 4A and 4C). However, all of the four *cecropin* genes had a sub-peak at 6 h after fungal infection, and tended to decrease later, to 36 h. From 36 to 48 h, the highest induced expression levels were reached (Fig. 4A). All four *gloverin* genes also reached a peak at 48 h after fungal infection, but without the sub-peak seen in *cecropin* genes at 6 h (Fig. 4C).

In *moricin* family, the expression pattern of each gene induced by *E. coli* was similar to that of the fungus *P. pastoris* (Fig. 4B). Both induced peaks for *Bmmor* were seen at 8 h after *E. coli* or *P. pastoris* infection. However, induction of *Bmmor* by *S. aureus* was about half the level of induction by *E. coli* or *P. pastoris* (Fig. 4B). These differences in induced expression pattern may reflect different signaling pathways regulating the immune response to bacteria and fungi [6]. Interestingly, in *gloverin* family, the induced expressions of *Bmglv2–4* were significantly higher than *Bmglv1* in pupae infected by *E. coli*, *S. aureus*, and *P. pastoris* (Fig. 4C).

**Figure 5:**
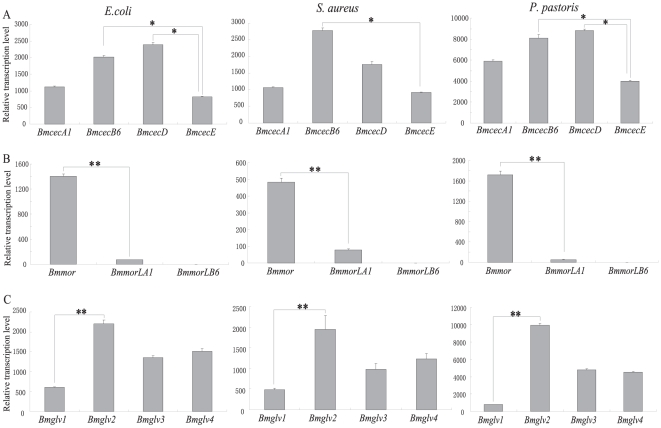
The average transcription activities of AMP gene families in *B. mori* induced by various microbes. The average values were calculated using the values in nine time points after immune challenge. Differences were significant at *P<0.001 or **P<0.0001 among each gene family.

To compare induced transcriptional activity, we calculated the average values for induced expression by time course and analyzed them using a *t*-test (Fig. 5). Of four *cecropin* genes, *BmcecB6* representing B subtype and *BmcecD* had the strongest induced activity, while *BmcecE* was the lowest (Fig. 5A). In *moricin* family, the induced activity of *Bmmor* was remarkably higher than *BmmorLA1* and *BmmorLB6* (Fig. 5B). *BmmorLB6* had scarcely any induced expression after microbe injection (Fig. 4B and 5B). Of the four *gloverin* genes, *Bmglv2* was significantly induced by all three microbes, with higher level than the others (Fig. 5C). The induced activity of *Bmglv3* was very similar to *Bmglv4* after infection by each microbe (Fig. 5C), while *Bmglv1* had the lowest value.

By comparative analysis of induced expression patterns within each gene family, major induced genes such as *BmcecB6*, *BmcecD*, *Bmmor* and *Bmglv2* were found to be significantly higher than other paralogs. The other genes in each family were induced at relatively low levels by each microbe. These results maybe indicated that these genes are highly induced as the major effector genes in the antimicrobial response, while the others play supporting roles.

### Relationship between antimicrobial activity and induced expression of AMPs

A possible relationship was explored combining the induced expression levels with the antimicrobial activities. The induced expression levels of *cecropin* and *moricin* families might be positively correlated with the antimicrobial activities of their AMPs. *BmcecB6*, *BmcecD* and *Bmmor* were induced with higher levels than other paralogs. Their recombinant AMPs also had wider antimicrobial spectrum and stronger antimicrobial activities within *cecropin* and *moricin* families (Fig. 3A, 3B, 4 and 5). Those AMPs encoded by genes with low induced expression, such as *BmcecE* and *BmmorLA1*, had limited antimicrobial spectrum and weak antimicrobial activities.

An exception was the *gloverin* family. *Bmglv2–4* were induced by each microbe at the higher level (Fig. 4C and 5C), but its antimicrobial activities were weaker than Bmglv1 against seven tested bacteria (Fig. 3C and Table 1). *Bmglv1* was induced at lower levels than other paralogs (Fig. 4C and 5C), but its antimicrobial activities were relatively stronger (Fig. 3C and Table 1). A reason was used to explain for the higher induction of *Bmglv2–4* than *Bmglv1 in vivo*. *Bmglv1* has five introns, one more than *Bmglv2–4*. *Bmglv2–4* were produced by gene duplication from the ancestor *Bmglv1* and lost its fifth intron. The fifth intron of *Bmglv1* contains CF2 inhibitory elements, which inhibit the embryonic expression of *Bmglv1* [29]. Presumedly, the difference in the fifth intron may cause different induced expression of *Bmglv1–4* in pupae.

### Sequence divergence of AMP genes

The silkworm genome has 11 *cecropin* genes, 12 *moricin* genes, and 4 *gloverin* genes (Fig. S4 and S5). Most of the genes in each family showed typical clustering on the same chromosome [2], [3], suggesting that these gene families were formed by gene expansion during evolution. For example, the *gloverin* family was formed by three gene duplication events from the ancestral gene *Bmglv1*, which produced three new genes, *Bmglv2–4* [29]. The *moricin-*like A and B subtypes were expanded from the ancestral gene, and had remarkable sequence divergence. Presumably, *BmmorLA2* and *BmmorLB6* initially arose from *Bmmor* and the A and B subfamilies were formed by 2-to-7 gene duplication events through unequal crossing over at the last few million years [2]. The expansion of the *cecropin* family was complicated. The cecropin B subtype contains six paralogs that are remarkably similar at the nucleotide level, indicating that they recently underwent gene duplication. The events occurred either between *BmcecD* and *BmcecE*, or between *BmcecA* and *BmcecC*, but it is unclear which was the ancestral gene for the two groups [2], [3].

The AMPs display high-level divergence in sequences and responses to pathogen challenges [37]. By above analysis, the antimicrobial activities of cecropins and moricins were remarkably different within each family, while four gloverins were similar (Fig. 3 and Table 1). This functional diversity may be the result of sequence divergence during evolution. To investigate the diversification of AMP families, we calculated synonymous and non-synonymous substitutions in each gene family using MEGA 3.0 (Table 2). The overall mean for nucleotide substitution per nonsynonymous site to substitution per synonymous site (*d_N_/d_S_*) was 0.29 for *cecropin* family, 0.426 for *moricin* family, and 0.15 for *gloverin* family. The values for *moricin* and *cecropin* families were obviously larger than *gloverin* family, suggesting that the two families underwent lower selection pressure, or faster evolution than *gloverin* family. That the two AMP families acquired more significant functional diversification might be important. The *gloverin* family might have undergone stabilizing selection in evolution. The four paralogs have the same antimicrobial spectrum, and only subtle differences in antimicrobial activities (Fig. 3 and Table 1).

**Table 2:** Summary of statistics on substitution patterns among AMP gene family in the silkworm.

Family	Region	No. of codons	*N_d_*	*S_d_*	*d_N_*	*d_S_*	*d_N_/d_S_*
Cecropin	All	58	124	50	0.222±0.164	0.765±0.531	0.290
	Signal	22	46	20	0.298±0.246	0.593±0.462	0.503
	Mature	36	78	30	0.187±0.145	0.806±0.673	0.232
Moricin	All	61	131	52	0.369±0.331	0.866±0.716	0.426
	Signal	21	46	17	0.417±0.380	0.893±0.950	0.467
	Mature	40	85	35	0.348±0.315	0.855±0.709	0.407
Gloverin	All	168	360	144	0.100±0.040	0.668±0.114	0.150
	Signal	37	77	34	0.190±0.070	0.725±0.308	0.262
	Mature	131	283	110	0.077±0.038	0.660±0.086	0.117

When natural selection drives rapid divergence of AMPs, the mature peptide is much less conserved than the signal peptide [38]. We calculated the *d_N_* and *d_S_* values in the segments of signal and mature peptides. The *d_N_/d_S_* ratios were 0.467, 0.503 and 0.262 for signal peptides, and 0.407, 0.232 and 0.117 for mature peptides of *moricin*, *cecropin* and *gloverin* families, respectively. These results indicated the signal peptides were slightly more variable than the mature peptides, suggesting that the mature peptides underwent stronger stabilizing selection than the signal peptides. A previous report on *Drosophila* antifungal peptides in the *drosomycin* family found similar results with signal peptides slightly more variable than mature peptides [4].

## Discussion

In the present study, we used the pET-32a(+) and pET-21d(+) expression systems to produce the recombinant proteins of three AMP families. They were expressed with mainly intracellular soluble forms in *E. coli* Rosetta™ (DE3) (Fig. S1). Directly, they were collected from the lysis supernatants and purified by Ni-NTA chromatography and by Sephadex G-10 chromatography. The recombinant cecropins and moricins contained the fragment of Trx(105aa)-His·Tag (6aa)-Thrombin-S·Tag in the N terminal. After digested by EK and purified by twice molecular ultrafiltrations. The molecular weights of purified cecropins (Fig. 2A) and moricins (Fig. 2B) were 4.0 kD and 4.7 kD, respectively. In their N terminal, the EK-digested cecropins and moricins only contain two extra residues (Ala-Met), which have little impact for their activity [39]. The gloverins contained the 8aa fragment of Leu-Glu-6His·Tag in the C terminal, carrying two extra amino acids (Leu-Glu) by *Xho* I digestion (Fig. 1C). In a previous study, a recombinant progloverin-His of *Trichoplusia ni* contained a His tag in the C terminal and had stronger antimicrobial activities [34]. It is suggested that the His tags of recombinant silkworm gloverins in their C terminal did not reduce their antimicrobial activity. In addition, about the concentration of recombinant gloverins, previous studies have reported that 20 µg recombinant Bmglv1–4 (10 µM) showed obvious antimicrobial activity against *E. coli* [28], [33]. In this study, we used the 5 µM recombinant AMPs to test their antimicrobial activities and found they have antimicrobial function (Fig. 3).

The evolution and functional divergence of AMPs has focused on the battle between hosts and pathogens. In the silkworm, AMPs are encoded by multiple gene families, such as *cecropin* and *moricin*. Their biological functions showed remarkable diversity, by antimicrobial spectrum and antimicrobial activity (Fig. 3 and Table 1). Therefore, we propose that major effector genes became prominent among the AMP family during evolution. BmcecB6, BmcecD and Bmmor have stronger antimicrobial activity and/or a wider antimicrobial spectrum than other paralogs in the same family. In a previous study, we also found that Drosomycin might play a major role in the *Drosophila* immune response because of its broader antifungal spectrum and higher induction activity than other paralogs of in the *drosomycin* family [23], [24].

Generally, AMP genes are strongly and quickly induced when hosts are infected by invaders. The silkworm AMP genes were also rapidly induced in larvae fat bodies injected with lipopolysaccharide or microbes [2], [3]. In this study, the systemic comparison of the induction patterns of three gene families revealed significant induction characteristics when day-2 pupae were infected with three different types of microbes. The most effective genes with the strongest antimicrobial activity, BmcecB6, BmcecD and Bmmor, had the highest level of induction, suggesting that high induction is a hallmark of the most effective AMP genes in the antimicrobial response. The distinct induction levels were closely related to regulatory elements located in the upstream region or intron [6]. A previous bioinformatic analysis suggested that the number and location of regulatory elements are likely to be crucial for regulating the levels of AMP genes [2]. The induced expression levels in this study provided indirect experimental evidence for an explanation of the obvious differences among these AMP genes of *cecropin* and *moricin* families.

In the upstream regulatory region of the *cecropin* family, *BmcecBs* and *BmcecD* have concentrated cluster elements of three NF-κB like elements, and one GATA element, while the distribution of these two kinds of elements is discrete and far from the transcription site in the other paralogs. This suggested that the cluster is similar to an enhancer for up-regulating the expression of *BmcecBs* and *BmcecD*. In the *moricin* family, the discrete distribution of regulatory elements might cause the weak transcriptional activity of *BmmorLA1* and *A2*. The *BmmorLB* subtype had fewer and more discrete sites than *BmmorLAs*. In contrast, two NF-κB-like, and three GATA elements were found within 200 bp upstream of the *Bmmor* start codon. This might be why the induced activity of *Bmmor* was higher than *BmmorLAs* and *BmmorLBs*.

In the *gloverin* family, the recombinant Bmglv1 had higher antimicrobial activity than recombinant Bmglv2–4, but its induction was the weakest of the paralogs. In contrast, the antimicrobial activities of recombinant Bmglv2–4 were relative weaker, but their induced expressions were significantly higher than *Bmglv1*. A study by Mrinal and Nagaraju [29] indicated that *Bmglv2–4* were produced from *Bmglv1* by gene duplication, and lost the fifth intron in the 3′-untranslated region of *Bmglv1*. The missing intron contains a CF2-binding site and acts as a repressor for inhibiting *Bmglv1* expression in embryonic stage. Chorion factor 2 (CF2) is a typical zinc finger factor. In *Drosophila*, CF2 factor not only works in embryo stage, but also in other developmental stages (FlyBase). The homologs of *Drosophila* CF2 factors were found in the silkworm genome and were expressed in the pupae stage. Therefore, we proposed that the loss of the fifth intron caused the higher induction of *Bmglv2–4*. In other words, the selective pressure of intron loss was to achieve embryonic expression, and to enhance the induction of transcription during bacterial infection.

Interestingly, we also found that these AMP genes were higher induced by fungus than bacterium. Especially in *cecropin* and *gloverin* families, the induced peak values by *P. pastoris* infection were 5–10 times higher than *E. coli* or *S. aureus* (Fig. 4). It is suggested they be likely to restrain the growth of fungi. However, most previous reports have indicated that these three families from the silkworm or other insects were typical antibacterial peptides, which have no antifungal activity or very weak. For example, cecropins had strong antibacterial activity against Gram-netative and Gram-positive bacteria [40], [41], [42], [43]. Only weak antifungal activity of cecropin A was found [10], [41]. Moricin isolated from the silkworm hemolymph had greatly strong antibacterial activity against Gram-netative and Gram-positive bacteria [25]. Moricins from *M. sexta* and *Spodoptera litura* also showed strong antibacterial activity, but not antifungal activity [44], [45]. Gloverin was first isolated from pupae of the giant silk moth *Hyalophora Cecropia* [33], thereafter, its homologs were found in *Helicoverpa armigera* [35], *T. ni* [34], *M. sexta* [44] and *Galleria mellonella* [46]. In these studies, Gloverin only had anti-negative-bacterial activity [28], [33], [35], [36]. Our results found four silkworm gloverins had obvious antimicrobial activity against two Gram-positive bacteria (*B. thuringiensis* and *B. thuringiensis subsp. Galleriae*), besides five Gram-negative bacteria (Fig. 3C). We also tested the antifungal assays of Bmglv1 and Bmglv2 using MICs method against *P. pastoris*. Unfortunately, they could not significantly inhibit the growth of *P. pastoris* (Fig. S6). Summarily, it is very interesting that these AMP genes were strongly induced but not active against *P. pastoris*. We hypothesized that their induced transcription may be associated with pattern recognition and signaling pathway.

The evolution of AMP genes is a model for adaptive evolution. AMPs in the silkworm and other insects, in contrast to organisms with adaptive immune systems, serve as the primary microbial- and fungal-killing proteins, and may be particularly important for preventing infection by non-coevolving saprophytic organisms. Furthermore, a large number of AMP genes of *cecropin*, *moricin* and *gloverin* families were induced to express in high systemic levels after infection (Fig. 4 and 5). Based on our results of *cecropin* and *moricin* families presented in this study and *drosomycin* family in *D. melanogaster* in the previous study [23], [24], we seem to found a connection of major genes between their strong antimicrobial activities and high induced expression levels in these AMP families. On second thoughts, we suppose that the AMPs of cecropins and moricins in *B. mori* and drosmycins in *D. melanogaster* have biological functions at three hierarchical levels in the antimicrobial response. Most AMP genes have low basic expression in the non-infected status [2], which may constitute the primary immune barrier. When the host is attacked by microbes, the major AMP genes, such as *BmcecB6*, *BmeceD*, *Bmmor* and *Drs*, were rapidly induced at very high levels. They formed the second line of the immune barrier that is crucial for eliminating infection. The other antimicrobial peptides with weak induced expression, weak antimicrobial activities and narrow antimicrobial spectrum may have functions as backups for the major AMPs as tertiary immune barrier.

The phenomenon examined here has probably been formed by long-term evolutionary selection, which introduced functional divergence among the AMP families and even within their paralogs, and established the broad antimicrobial spectrum of the immune system. The multi-level barriers, involving major and complementary AMPs that efficiently kill different invasive microbes by multi-target attacking, achieve successful defense through a multi-level, comprehensive, rapid, economic, and effective immune model of AMPs. For the present, only three AMP families of cecropins and moricins in *B. mori* and drosmycins in *D. melanogaster* may support this viewpoint. Whether the phenomenon of major effector genes is a common hallmark of insect AMPs or not, further investigation needed to be done will be helpful in understanding the evolution of insect immune system.

## Materials and Methods

### Insects and microorganisms

*B. mori* strain *Dazao* was from the Sericulture & Agri-Food Research Institute, Guangdong Academy of Agricultural Sciences, China. *E. coli* Rosetta™(DE3) strain was used to express recombinant AMPs. *S. aureus*, *E. coli* (K_12_D_31_ strain) and *P. pastoris* were used to infect silkworm pupae to test inducible expression. To test antimicrobial activity, the following microbes were used: five Gram-positive bacteria: *S. aureus*, *B. bombysepticus*, *B. subtilis*, *B. thuringiensis* and *B. thuringiensis subsp. galleriae*; five Gram-negative bacteria: *E. coli* (K_12_D_31_ strain), *S. marcescens*, *P. aeruginosa*, *X. pv. campestris* and *R. dolaanacearum*.

### Cloning AMP genes of the silkworm

In the silkworm genome, the *cecropin*, *moricin* and *gloverin* families contain 11, 12 and 4 paralogs, respectively. These paralogs are very similar at the nucleotide level within each family. For the three families, five *cecropin* (A1, B6, C, D, E), five *moricin* (A1, LA1, B1, B5, B6), and four *gloverin* (*Bmglv*1–4) genes were cloned. *Moricin* from *M. sexta* (*Msmor*) was cloned as a control [32]. For cloning target genes, specific primers were designed to amplify the gene segments for *Bmcec* (A1, B6, C, D, E) and *Bmglv1* (Table S1). Because of high similarity in the *gloverin* family, specific forward primers for *Bmglv*2 and *Bmglv3* were designed, while a degenerate forward primer was designed for cloning *Bmglv4*, and common reverse primer was used for Bmglv 2–3 (Table S1). For the *moricin* family, three overlapping and complementary primers were designed to amplify the chosen *moricin* paralogs (Table S1). The *cecropin* and *gloverin* family genes were amplified from the cDNA of the pupae infected by *E. coli*. The *moricin* paralogs were synthesized directly by PCR, using overlapping and complementary primers.

### Construction of recombined expressive vectors and recombinant expression

The PCR products of all target genes were purified with the EZNA Cycle-Pure Kit (Omega, USA) and digested with *Nco* I and *Xho* I. The digested products of *cecropin* and *moricin* paralogs were ligated to pET-32a (+) vectors, which contain a thioredoxin (105 aa) and a 6-His tag. The digested products of *gloverin* paralogs were ligated to pET-21d (+) vectors, which only contain a 6-His tag. The recombinant expression vectors containing target genes were extracted as above, and sequenced in an ABI 377 automated sequencer. All vectors were used to transform *E. coli* Rosetta (DE3). After host cells containing recombinant expression vectors in LB-medium at 37°C reached an optical density (OD) of 0.5–0.8 at 600 nm, isopropyl-beta-D-thiogalactopyranoside (IPTG) was added to a final concentration of 1 mM to induce bacterial production of recombinant proteins. Incubation continued for another 3–5 h at 37°C, and then the cells were centrifuged at 5,000–10,000 RPM for 2–5 min at 4°C.

### Purification of expression products

All recombinant cecropins, moricins and gloverins were expressed with mainly intercellular soluble form in *E. coli* Rosetta (DE3). Thus, the recombinant expression was not repressed by the toxicity of these proteins to *E. coli*. These recombinant proteins were collected directly from the supernatants and purified by Ni-NTA affinity chromatography using the 6-His tag, and Sephadex G-10 chromatography for desalination. The recombinant products of cecropin and moricin family will contain the fragment of Trx(105aa)-His·Tag (6aa)-Thrombin-S·Tag in the N terminal, whereas, the products of gloverin family will contain the 8aa fragment of Leu-Glu-6His·Tag in the C terminal, carrying two extra amino acids (Leu-Glu) by *Xho* I digestion.

Whereafter purified recombinant cecropins and moricins (100 µg/µl) were digested at 16°C for 16 h using 0.5 IU enterokinase to separate the target proteins from the Trx-His Tag-Thrombin-S Tag. The digested products were ultrafiltered twice. Thioredoxin was removed using the 10-kDa molecular weight cut-off ultrafiltration column. Filtrate was passed through a 3-kDa molecular weight cut-off ultrafiltration column to obtain 4.0-kDa cecropin peptides and 4.7-kDa moricin peptides, which contain two extra amino acids (Ala-Met) after EK digestion. These purified cecropins and moricins were tested by Tricine-SDS-PAGE gels (16.5% separating gel), while the purified gloverins with 8aa fragment of Leu-Glu-6His·Tag by Sephadex G-10 chromatography was tested by Tricine-SDS-PAGE gels (15% separating gel).

### Antimicrobial activity assay

Antimicrobial activity of purified expression products was assayed using the ultrasensitive radial diffusion method and the MICs method seeded with Gram-negative or Gram-positive bacteria. Briefly, 90 mm plates were poured with LB-medium seeded with the tested bacteria. Each sample (5 µmol/L, 10 µL) was dropped into a 2.7 mm pore and was repeated three repeats, and the plate was incubated at 37°C for 24 h. The size of the clear area around the bacteria was measured, as shown in Figure S3 for the moricin family. In the MICs method, the expression products of cecropin and moricin were diluted to final concentrations of 20, 10, 5, 2.5, 1.25, 0.625, 0.313 or 0.157 µmoL/L, and added to bacteria at logarithmic phase. For the gloverin family, eight grades of 2.5 and 1.25 µmoL/L were diluted to final concentrations of 2.5, 2.2, 2.0, 1.8, 1.6, 1.4, 1.2, or 1.0 µmoL/L. And then the bacteria were incubated at 30°C for 24 h. Absorbance at 595 nm was measured and was repeated three times, and the MICs determined when the average absorbance was equal to the control sample.

### RNA extract and quantitative real-time PCR

Total RNAs were isolated using TRIzol Reagent (Invitrogen) from the day-2 pupae collected at 2, 4, 6, 8, 12, 24, 36, 48 and 72 h after injection with *S. aureus*, *E. coli* (K12D31) or *P. pastoris*. Contaminating genomic DNA was digested with Rnase-free Dnase I (Promega) for 15 min at 37°C. The quality of extracted RNA was checked by agarose gel electrophoresis and quantified in a spectrophotometer. The first strand of cDNA was synthesized using M-MLV Reverse Transcriptase (TOYOBO). Real-time PCR reactions were carried out on cDNA samples using real-time PCR Master Mix (TOYOBO). Because of the high similar sequences in each gene family, we designed specific primers to distinguish paralogs in each of the gene families (Table S2). The cytoplasmic *actin* (No. X04507) of the silkworm was tested as a reference to normalize variance among different samples. The ABI Prism 7300 Sequence Detection System (Applied Biosystems) was employed for quantification. Data from quantitative real-time PCR were analyzed using the 2^−ΔΔC^_T_ Method [47]. Relative expression values were calculated after normalizing against the maximum expression value.

### Sequence analysis

Silkworm antimicrobial peptides were identified in previous studies [2]. Coding sequences were aligned using the ClustalX program and checked by hand. We adopted the method of Nei-Gojobori to look at patterns of nonsynonymous and synonymous substitutions among the sequences [48]. We calculated the overall *N_d_* and *S_d_*, and *d_N_* and *d_S_*, for signal and mature peptides of the *cecropin*, *moricin* and *gloverin* families.

## Supporting Information

Figure S1 **SDS-PAGE analysis of soluble fractions of the recombinant AMPs expressed in** ***E. coli*** **Rosetta™(DE3).** The arrows indicate the positions of expressed recombinant AMPs. A) Cecropin family: M: Protein molecular weight marker; 1: Bacterial lysate containing recombinant pET-32a (+)-AMPs plasmid; Lane 2, 4, 6, 8, 10, 12 and 14: Precipitate fractions of bacterial lysate from pET-32a(+)-*Bmcec-A1*(-*C*, -*E*, -*D1*, -*D2*, -*B6*, -*D*); Lane 3, 5, 7, 9, 11, 13, and 15: Supernatant fractions of bacterial lysate from pET-32a(+)-*Bmcec-A1*(-*C*, -*E*, -*D1*, -*D2*, -*B6*, -*D*). B) Moricin family: M: Protein molecular weight marker; 1: Bacterial lysate containing recombinant pET-32a (+)-AMPs plasmid; Lane 2, 4, 6, 8, 10, and 12: Precipitate fractions of bacterial lysate from pET-32a (+)-*Bmmor* (-*LA1*, -*LB5*, -*LB6*, -*LB1*) and pET-32a(+)-*Msmor*; Lane 3, 5, 7, 9, 11, and 13: Supernatant fractions of bacterial lysate from pET-32a(+)-*Bmmor* (-*LA1*, -*LB5*, -*LB6*, -*LB1*) and pET-32a (+)-*Msmor*. C) Gloverin family: M: Protein molecular weight marker; CK: Bacterial lysate containing pET-21d plasmid; Lane 1, 3, 5, 7, and 9: Supernatant fractions of bacterial lysate from pET-21d-Bmglv-1 (-4, -4i, -3, -2); Lane 2, 4, 6, 8, and 10: Precipitate fractions of bacterial lysate from pET-21d- Bmglv-1 (-4, -4i, -3, -2).
(PDF)

Figure S2 **SDS-PAGE analysis of the fusion proteins purified by Ni-NTA chelating Sepharose chromatogramphy.** A) BmcecB6 fusion protein. M: Protein molecular weight marker; 1: bacterial lyate containing pET-32a (+) expression vector; 2–3: washed from Ni-NTA chelating Sepharose chromatogramphy by Lysis buffer, 4–5: washed from Ni-NTA chelating Sepharose chromatogramphy by Wash buffer; 6–9: washed from Ni-NTA chelating Sepharose chromatogramphy by Elution buffer. B) Bmmor fusion protein. M: Protein molecular weight marker;1, bacterial lyate containing pET-32a(+) expression vector; 2–3,washed from Ni-NTA chelating Sepharose chromatogramphy by Lysis buffer; 4–5, washed from Ni-NTA chelating Sepharose chromatogramphy by Wash buffer; 6–8, washed from Ni-NTA chelating Sepharose chromatogramphy by Elution buffer. C) Bmglv4 fusion protein. M: Protein molecular weight marker; CK: bacterial lyate containing pET-32a(+) expression vector; 1: washed from Ni-NTA chelating Sepharose chromatogramphy by Lysis buffer; 2–3 washed from Ni-NTA chelating Sepharose chromatogramphy by Wash buffer; 4–7: washed from Ni-NTA chelating Sepharose chromatogramphy by Elution buffer.
(PDF)

Figure S3 **Antimicrobial activity assay of moricin family against the 10 testing microbes using ultra sensitive radial diffusion method.** Pores 1–6 on the LB-medium plates indicated the silkworm BmmorA1, BmmorLA1, *M. sexta* moricin, BmmorB1, BmmorB5 and BmmorB6, respectively. CK indicated sterile water as negative control. Each of test samples (5 µmol/L, 10 µL) was dropped into the 2.7 mm pore. The size of the clear area around the bacteria was measured after incubating at 37°C for 24 h.
(TIF)

Figure S4 **Sequence alignments of coding sequences for cecropin family (A), moricin family (B) and gloverin family (C).** The sequences were aligned using the ClustalX program. The dots indicate conserved nucleotide sites. The arrows indicate the digested sites of mature peptides.
(PDF)

Figure S5 **Sequence alignments of protein sequences for cecropin family (A), moricin family (B) and gloverin family (C).** The sequences were aligned using the ClustalX and regions of homology were highlighted using BoxShade (http://www.ch.embnet.org/software/BOX_form.html).
(PDF)

Figure S6 **Antifungal assay of Bmglv1 and Bmglv2.** The growth of fungi at 30°C with or without BmGlvs was detected by recording optical density at 570 nm. 0.1 M PBS buffer (pH 7.4) was used as a negative control. 45 uM of each BmGlvs was used in the experiments. The data show means ± the standard errors.
(PDF)

Table S1 **Cloning primers for recombinant expression of antibacterial peptides.**
(PDF)

Table S2 **Primers for quantitive real-time RT-PCR.**
(PDF)
