# Supplementary figures and images for: Functional Divergence among Silkworm Antimicrobial Peptide Paralogs by the Activities of Recombinant Proteins and the Induced Expression Profiles
Source: PLoS One. 2011 Mar 29;6(3):e18109. doi: 10.1371/journal.pone.0018109 (PMC3066212; doi:10.1371/journal.pone.0018109)

Figure S1

A

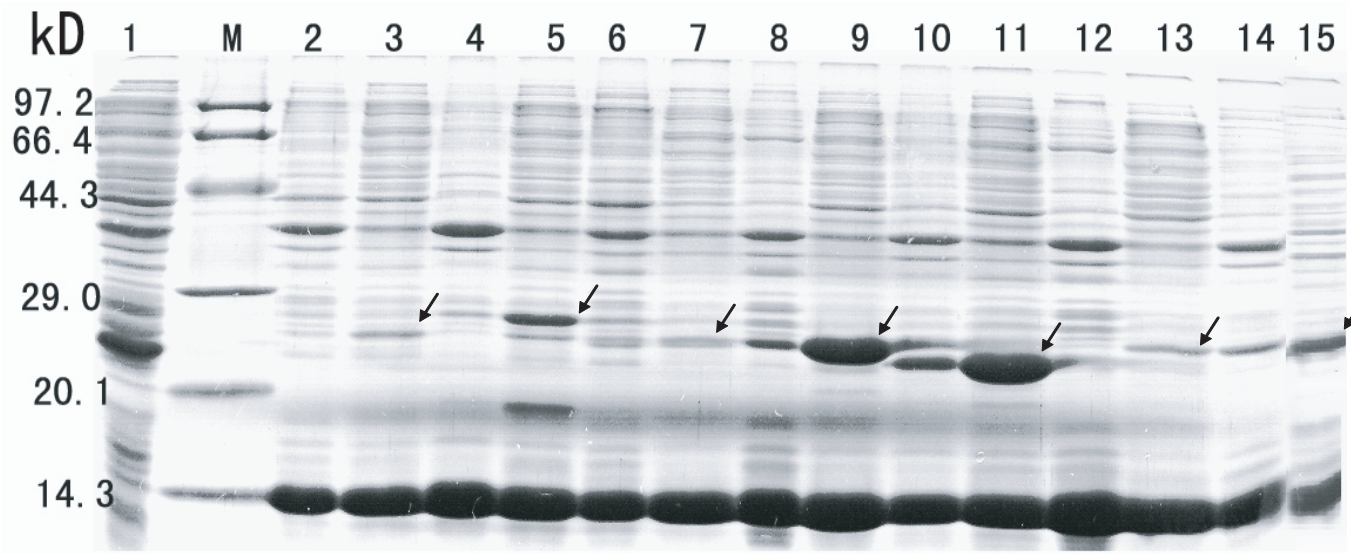

B

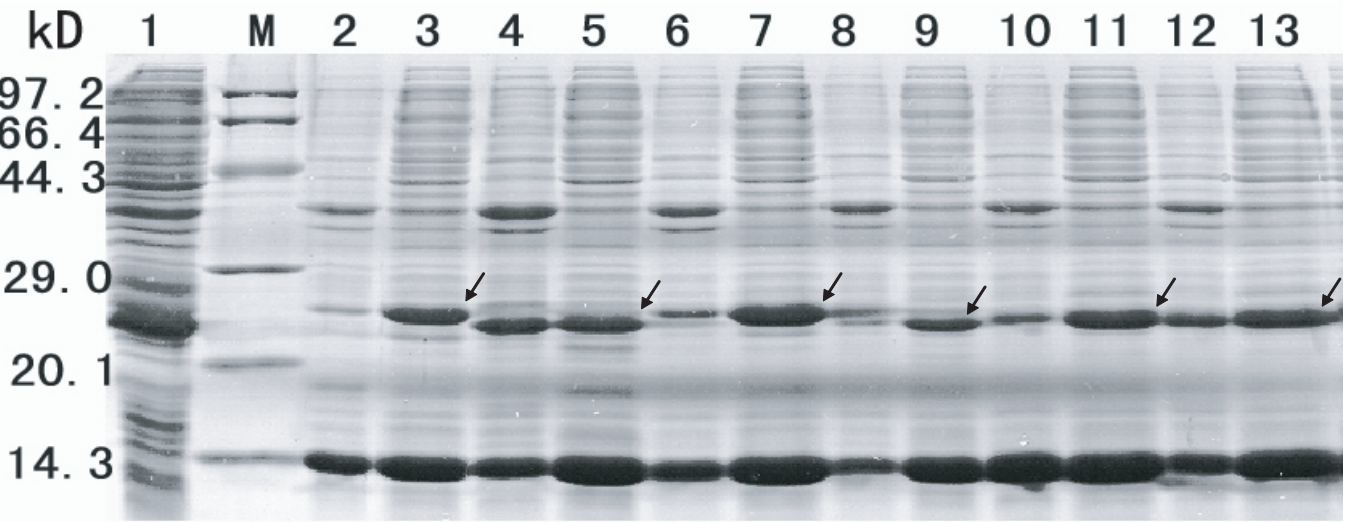

C

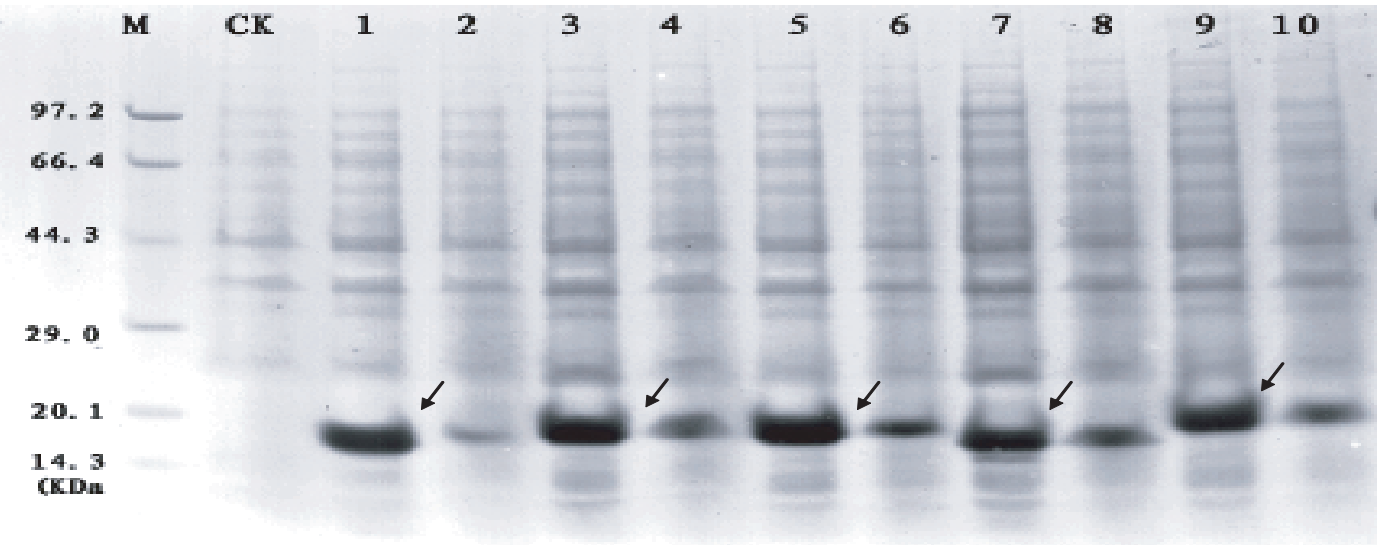

Supplement: Figure S1 — SDS-PAGE analysis of soluble fractions of the recombinant AMPs expressed in E. coli Rosetta™(DE3). The arrows indicate the positions of expressed recombinant AMPs. A) Cecropin family: M: Protein molecular weight marker; 1: Bacterial lysate containing recombinant pET-32a (+)-AMPs plasmid; Lane 2, 4, 6, 8, 10, 12 and 14: Precipitate fractions of bacterial lysate from pET-32a(+)-Bmcec-A1(-C, -E, -D1, -D2, -B6, -D); Lane 3, 5, 7, 9, 11, 13, and 15: Supernatant fractions of bacterial lysate from pET-32a(+)-Bmcec-A1(-C, -E, -D1, -D2, -B6, -D). B) Moricin family: M: Protein molecular weight marker; 1: Bacterial lysate containing recombinant pET-32a (+)-AMPs plasmid; Lane 2, 4, 6, 8, 10, and 12: Precipitate fractions of bacterial lysate from pET-32a (+)-Bmmor (-LA1, -LB5, -LB6, -LB1) and pET-32a(+)-Msmor; Lane 3, 5, 7, 9, 11, and 13: Supernatant fractions of bacterial lysate from pET-32a(+)-Bmmor (-LA1, -LB5, -LB6, -LB1) and pET-32a (+)-Msmor. C) Gloverin family: M: Protein molecular weight marker; CK: Bacterial lysate containing pET-21d plasmid; Lane 1, 3, 5, 7, and 9: Supernatant fractions of bacterial lysate from pET-21d-Bmglv-1 (-4, -4i, -3, -2); Lane 2, 4, 6, 8, and 10: Precipitate fractions of bacterial lysate from pET-21d- Bmglv-1 (-4, -4i, -3, -2). (PDF) [file pone.0018109.s001.pdf]

Figure S2

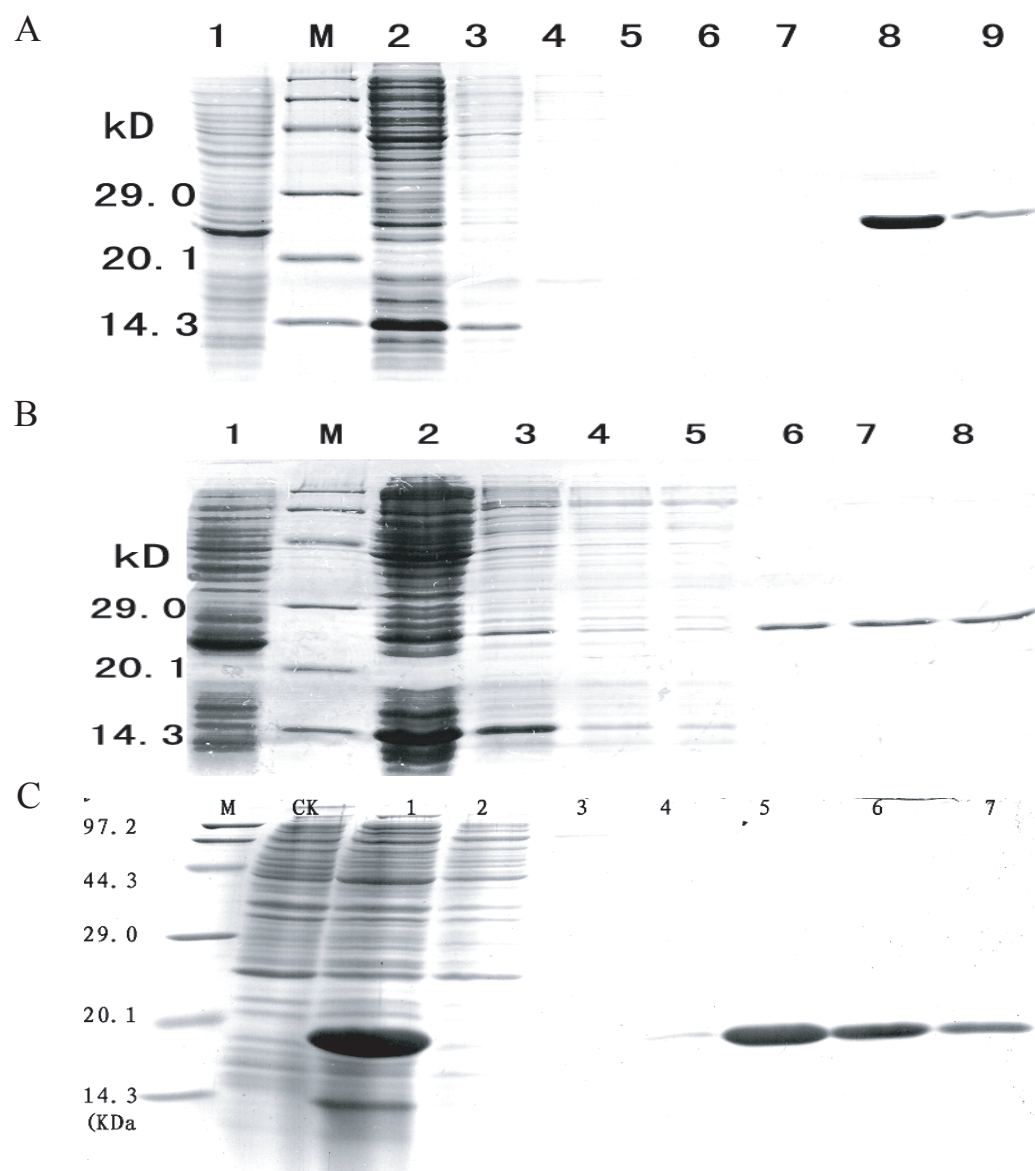

Supplement: Figure S2 — SDS-PAGE analysis of the fusion proteins purified by Ni-NTA chelating Sepharose chromatogramphy. A) BmcecB6 fusion protein. M: Protein molecular weight marker; 1: bacterial lyate containing pET-32a (+) expression vector; 2–3: washed from Ni-NTA chelating Sepharose chromatogramphy by Lysis buffer, 4–5: washed from Ni-NTA chelating Sepharose chromatogramphy by Wash buffer; 6–9: washed from Ni-NTA chelating Sepharose chromatogramphy by Elution buffer. B) Bmmor fusion protein. M: Protein molecular weight marker;1, bacterial lyate containing pET-32a(+) expression vector; 2–3,washed from Ni-NTA chelating Sepharose chromatogramphy by Lysis buffer; 4–5, washed from Ni-NTA chelating Sepharose chromatogramphy by Wash buffer; 6–8, washed from Ni-NTA chelating Sepharose chromatogramphy by Elution buffer. C) Bmglv4 fusion protein. M: Protein molecular weight marker; CK: bacterial lyate containing pET-32a(+) expression vector; 1: washed from Ni-NTA chelating Sepharose chromatogramphy by Lysis buffer; 2–3 washed from Ni-NTA chelating Sepharose chromatogramphy by Wash buffer; 4–7: washed from Ni-NTA chelating Sepharose chromatogramphy by Elution buffer. (PDF) [file pone.0018109.s002.pdf]

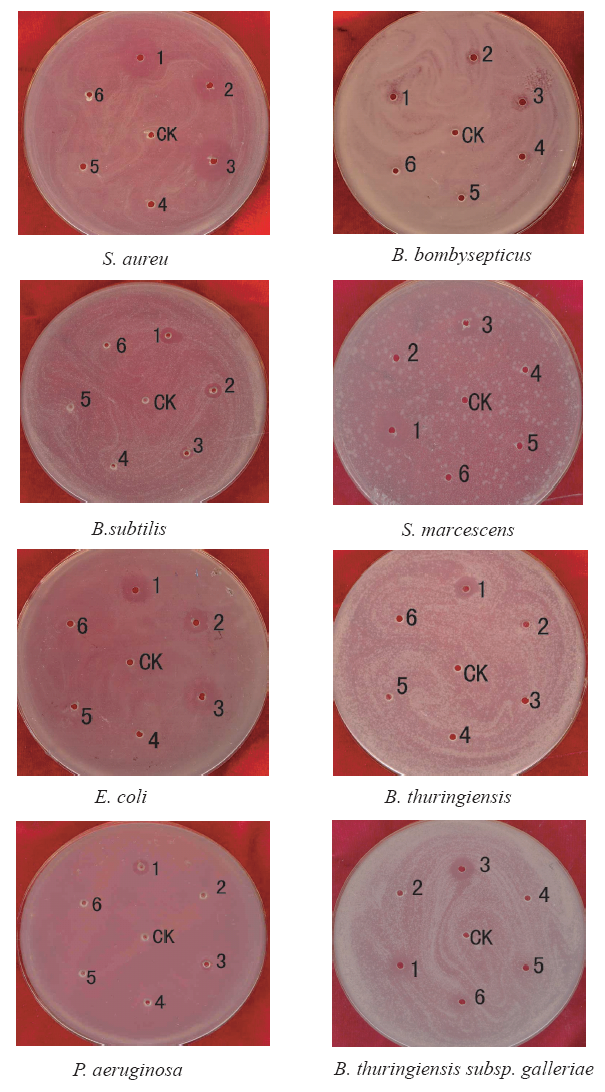

Supplement: Figure S3 — Antimicrobial activity assay of moricin family against the 10 testing microbes using ultra sensitive radial diffusion method. Pores 1–6 on the LB-medium plates indicated the silkworm BmmorA1, BmmorLA1, M. sexta moricin, BmmorB1, BmmorB5 and BmmorB6, respectively. CK indicated sterile water as negative control. Each of test samples (5 µmol/L, 10 µL) was dropped into the 2.7 mm pore. The size of the clear area around the bacteria was measured after incubating at 37°C for 24 h. (TIF) [file pone.0018109.s003.tif]

Figure S6

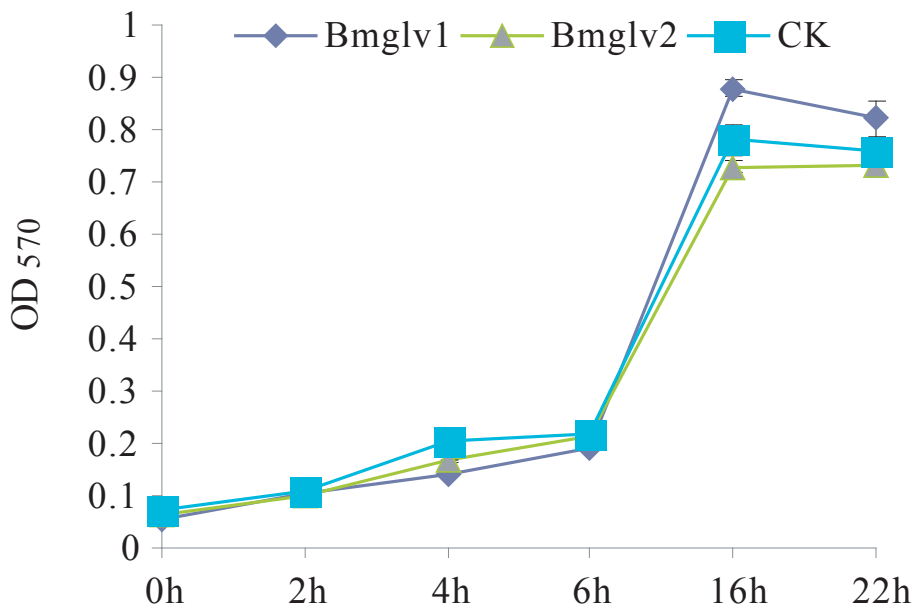

Supplement: Figure S6 — Antifungal assay of Bmglv1 and Bmglv2. The growth of fungi at 30°C with or without BmGlvs was detected by recording optical density at 570 nm. 0.1 M PBS buffer (pH 7.4) was used as a negative control. 45 uM of each BmGlvs was used in the experiments. The data show means ± the standard errors. (PDF) [file pone.0018109.s006.pdf]
