# Supplementary material for: Functional Divergence among Silkworm Antimicrobial Peptide Paralogs by the Activities of Recombinant Proteins and the Induced Expression Profiles
Source: PLoS One. 2011 Mar 29;6(3):e18109. doi: 10.1371/journal.pone.0018109 (PMC3066212; doi:10.1371/journal.pone.0018109)
Supplement: Figure S4 — Sequence alignments of coding sequences for cecropin family (A), moricin family (B) and gloverin family (C). The sequences were aligned using the ClustalX program. The dots indicate conserved nucleotide sites. The arrows indicate the digested sites of mature peptides. (PDF) [file pone.0018109.s004.pdf]

Figure S4

A

```
BmcecA1 ATG AAT TTC GTA CGT ATT TTG AGC TTC GTC TTC GCG TTG GTG CTG GCT CTC GGC GCG GTC AGC GCT GCT CCT GAG CCC
BmcecA2 ... ... ..T ... ... ... ... ... ... ... ... ... ... ... ... ... ... ... ... ... ... ... ... ..C ... ...
BmcecB1 ... ... ... .C. AAG ..C C.A TC. ... ... ... ..T C.. ... ... ... T.G A.. AT. AC. ... ... ... ..C ..A ...
BmcecB2 ... ... ... .C. AAG ..C C.A TC. ... ... ... ..T C.. ... ... ... T.G A.. AT. AC. ..T ... ... ..C ... ...
BmcecB3 ... ..C ... .C. AAG ..C C.A TC. ... ... ... ..T C.. ... ... ... T.G A.. AT. AC. ... ... ... ..C ... ...
BmcecB4 ... ... ... .C. AAG ..C C.A TC. ... ... ... ..T C.. ... ... ... T.G A.. AT. AC. ... ... ... ..C ... ...
BmcecB5 ... ... ... .C. AAG ..C C.A TC. ... ... ... ..T C.. ... ... ... T.G A.. AT. AC. ... ... ... ..C ... ...
BmcecB6 ... ... ... .C. AAG ..C C.A TC. ... ... ... ..T C.. ... ... ... T.G A.. AT. AC. ... ... ... ..C ... ...
BmcecC  ... ..C ..T ... AAG ..C C.. T.. G.. ... ..A ... C.C A.. ... ... T.G A.. AT. .CG ..T ..C ..G .T. ..A ...
BmcecD  ... ..A ... TCG AAA ... ..C GTT ... ..G ... ..T A.T ..T T.C ..C ACG .CT T.. ... TCG ..A ... ..C --- ---
BmcecE  ... ... ... TC. A.A GC. C.. TTT .A. ..T ... ..C G.. T.T ... .TG TGT .CG AGC ..T .TG ..C ... ..G ..A ..G

BmcecA1 AGG TGG AAA CTC TTC AAG AAA ATT GAG AAA GTG GGA CGC AAC GTT CGC GAT GGA TTA ATC AAA GCG GGT CCA GCT ATA
BmcecA2 ... ... ..G ... ... ... ... ... ... ... ... ... ... ... ... ... ... ... ... ... ... ... ... ... ... ...
BmcecB1 ... ... ..G A.. ... ... ... ... ..A ... A.. ..C A.G ... A.C ..T ... ..C A.C G.. ... ... ..C ..G ..G ..C
BmcecB2 ... ... ..G A.. ... ... ... ... ..A ... A.. ..C A.G ... A.C ..T ..C ..C A.C G.. ... ... ..C ..G ..G ..C
BmcecB3 ... ... ..G A.. ... ... ... ... ..A ... A.. ..C A.G ... A.. ..T ..C ..C A.C G.. ... ... ..C ..G ..G ..C
BmcecB4 ... ... ..G A.. ... ... ... ... ..A ... A.. ..C A.G ... A.C ..T ..C ..C A.C G.. ... ... ..C ..G ..G ..C
BmcecB5 ... ... ..G A.. ... ..A ... ... ..A ... A.. ..C A.G ... A.C ..T ..C ..C A.C G.. ... ... ..C ..G ..G ..C
BmcecB6 ... ... ..G A.. ... ... ... ... ..A ... A.. ..C A.G ... A.C ..T ..C ..C A.C G.. ... ... ..C ..G ..G ..C
BmcecC  .AA C.. ..G G.. ... ..A .T. ... ... ..G A.T ..T ... ..T ..C ... .G. ... G.C ..T .CG ..A ..A ... ..C G.G
BmcecD  --- G.C ..C T.. ... ... G.T C.. ..A ... A.. ..T .AG .GG ... ..A ..C .CC G.C ... .GC ... .C. ... ..A G.C
BmcecE  ..A ... ..G A.T ... ... ... ..C ..A ..G ... ..T .AG ... A.. ..T ... ..G A.. ... ..G ..T ..A ... ... G.C

BmcecA1 GCC GTC ATA GGG CAA GCG AAA TCC TTA GGA AAA TAG --- ---
BmcecA2 ... ... ... ... ... ..A ... ..A ... ... ..G ... --- ---
BmcecB1 .AG ... C.C ..T TCG ..T ... G.T A.. ... ... .GA --- ---
BmcecB2 .AG ... C.C ..T TC. ..T ... G.. A.. ... ... .GA --- ---
BmcecB3 .AG ... C.C ..T TCG ..T ... G.. A.. ... ... ..A --- ---
BmcecB4 .AG ... C.C ..T TCG ..T ... G.T A.. ... ... .GA --- ---
BmcecB5 .AG ... C.T ..T TCG ..T ... G.T A.. ... ... .GA --- ---
BmcecB6 .AG ... C.C ..T TCG ..T ... G.T A.. ... ... .GA --- ---
BmcecC  .TG ... G.T ..A ... ... GC. ... G.C ... .TG .AA --- ---
BmcecD  .A. AC. C.G .CA A.. ..A ... G.T C.C ... C.. GGA TAG ---
BmcecE  ..G ..G G.. ... ..G ... GCG A.. A.C .CT C.C GG. AAA TAA
```

B

```
Bmmor   ATG AAT ATT TTA AAA CTT TTC TTG GTT TTT ATG GTG GCA ATG TCT CTG GTG TCA TGT --- --- AGT ACA GCC GCT CCA
BmmorA1 ... G.C T.C C.T ..G TA. ... A.T ... G.A C.. ..A ..C C.. ..A T.. A.. GTT ..C --- --- ... GG. CAG ... GAT
BmmorA2 ... T.C T.C C.T ..G TA. ... A.T ... G.A C.. ..A ..C C.. ..A T.. A.. ATT ..C --- --- ... GGG CAG ... GAT
BmmorA3 ... T.C T.C C.T ..G TA. ... A.T ... G.A C.. ..A ..C C.. ..A T.. A.. ATT ..C --- --- ... GG. CAG ... GAT
BmmorB1 ... ..G G.A ..C .GT T.C ... .GC ..G G.G C.. .C. ATG C.C GTC ..C A.A ATG G.C GGA ACT ..C G.G ... C.. GA.
BmmorB2 ... ..G G.G ..C .GT ..A ... .GC ..G G.G C.. .C. ATG C.C GTC ..C A.A ATG G.C GGA ACT ..C G.G ... C.. GA.
BmmorB3 ... ..G G.A ..C .GT T.C ... .GC ..G G.G C.. .C. ATG C.C GTC ..C A.A ATG G.C GGA ACT ..C G.G ... C.. GA.
BmmorB4 ... ..G G.G ..C .GT A.A ... .GC ..G G.G C.. .C. ATG C.C GTC ..C A.A ATG G.C GGA ACT ..C G.G ... C.. GA.
BmmorB5 ... ..G G.G ..C .GT ..A ... .GC ..G G.G C.. .C. ATG C.C GTC ..C A.A ATG G.C GGA ACT ..C G.G ... C.. GA.
BmmorB6 ... ..G G.G ..C .GT A.A ... .GC ..G G.G C.. .C. ATG C.C GTC ..C A.A ATG G.C GGA ACT ..C G.G ... C.. GA.
BmmorB7 ... ..G G.A ..C .GT T.C ... .GC ..G G.G C.. .C. ATG C.C GTC ..C A.A ATG G.C GGA ACT ..C G.G ... C.. GA.
BmmorB8 ... ..G G.A ..C .GT T.C ... .GC ..G G.G C.. .C. ATG C.C GTC ..C A.A ATG G.C GGA ACT ..C G.G ... C.. GA.

Bmmor   GCA AAA ATA CCT ATC AAG GCC ATT AAG ACT GTA GGA AAG GCA GTC GGT AAA GGT CTA AGA GCC ATC AAT ATC GCC AGT
BmmorA1 C.G ... ..T ... G.. ..A AGT C.G ... .AG .GT ... ..A ATT A.T .CC ... ..C T.T .A. .TT C.. .CC GCA ..G G.A
BmmorA2 C.G ... ..T ... G.. ..A AGT C.G ... .AG .GT ..G ..A ATT A.T .CC ... ..C T.T .A. .TT C.. .CC GCA ..G G.A
BmmorA3 C.G ... ..T ... G.. ..A AGT C.G ... .AG .GT ..G ..A .TT A.T .CC ... ..C T.T .A. .TT C.. .CC GCA ..G G.A
BmmorB1 C.T ..G GGC ATC GGG ..A AT. ..C .GA .AG .GC ..G ..A .TT A.T AAA C.T ..A ..T .CC ..A ..A GGC G.A .GA GC.
BmmorB2 C.T ..G GGC ATC GGG ..A AT. ..C .GA .AG .GC ..G ..A .TT A.T AAA C.T ..A ..T .CC ..A ..A GGC G.A .GA GC.
BmmorB3 C.T ..G GGC ATC GGG ..A AT. ..C .GA .AG .GC ..G ..A .TT A.T AAA C.T ..A ..T .CC ..A ..A GGC G.A .GA GC.
BmmorB4 C.T ..G GGC ATC GGG ..A AT. ..C .GA .AG .GC ..G ..A .TT A.T AAA C.C ..G ..G .CC .T. ... GGC G.. .GG GCC
BmmorB5 C.T ..G GGC ATC GGG ..A AT. ..C .GA .AG .GC ..G ..A ATT A.T AAA C.T ..G ..G .CC .T. ... GGC G.. .GG GCC
BmmorB6 C.T ..G GGC ATC GGG ..A AT. ..C .GA .AG .GC ..G ..A .TT A.T AAA C.T ..A T.. .CC .TA ... GGC G.T .GA GCC
BmmorB7 C.T ..G GGC ATC GGG ..A AT. ..C .GA .AG .GC ..G ..A .TT A.T AAA C.T ..A ..T .CC ..A ..A GGC G.A .GA GC.
BmmorB8 C.T ..G GGC ATC GGG ..A AT. ..C .GA .AG .GC ..G ..A .TT A.T AAA C.T ..A ..T .CC ..A ..A GGC G.A .GA GC.

Bmmor   ACA GCC AAC GAT GTT TTC AAT TTC TTG AAA CCG AAG AAA AGA AAG CAT TAA
BmmorA1 ... ... C.T ..A ..C .AT .GC CA. G.. CGT AAC CGT GGG .AT C.A GG. ...
BmmorA2 ... ... C.T ..A ..C .AT .GC CA. G.. CGT AAC CGT GG. .AT C.A GG. ...
BmmorA3 ... ... C.T ..A ..C .AT .GC CA. G.. CGT AAC CGT GGG .AT C.A GG. ...
BmmorB1 G.C .GA C.. ..G ..C .A. C.A GA. .C. ... AAC .GC GG. T.. --- --- ---
BmmorB2 G.C .GA C.. ..G ..C .A. C.A GA. .C. ... AAC .GC GG. T.. --- --- ---
BmmorB3 G.T .GA C.. ..G ..C .A. C.A GA. .C. ... AAC .GC GG. T.. --- --- ---
BmmorB4 G.T .GA C.. ..C .CC .A. C.A CAG .C. C.G AAC .GC GG. T.. --- --- ---
BmmorB5 G.C .GA C.. ..C .CC .A. C.A CAG .C. C.G AAC .GC GG. T.. --- --- ---
BmmorB6 G.. .GA C.T ..A ..C .A. C.A GAG .CA ..G AAC .GC GGC T.. --- --- ---
BmmorB7 G.C .GA C.. ..G ..C .A. C.A GA. .C. ..A AAC .GC GG. T.. --- --- ---
BmmorB8 G.C .GA C.. ..G ..C .A. C.A GA. .C. ..A AAC .GC GG. T.. --- --- ---
```

C

```
Bmglv1 ATG TAT TCC AAG GTG TTG --- TTA TCC GCT GCA CTC CTT GTA TGC GTG AAC GCT CAA GTT TCT ATG CCT CCT GGT TAC
Bmglv2 ... A.. ..A ..T C.. ..T TAT A.C .T. ... A.. ACT ..G ..G ..T ..T ... ..A G.. ... .AC GGA ... T.. .A. ...
Bmglv3 ... A.. ... ..A T.. C.. TTT ..C AT. ..C A.. G.. ..G ..G ..T ..A ... ..A G.. ..A .AC .G. T.. T.. .A. ..T
Bmglv4 ... A.. ... ..A C.A ..A TAT ..C .T. ..C A.G G.. ..G ..G ..T ..C ... ..A G.. ... .A. --- --- T.G .AG ...

Bmglv1 GCA GAG AAG TAT CCG ATC ACC AGC CAA TTT TCA AAG TCA GTC CGA CAC CCT CGC GAT ATT CAC GAC TTT GTC ACT TGG
Bmglv2 ... ..A G.T ..C T.. ... .G. G.G ... .CC ... .G. --- --- ... ... ..C ..T ..C --- --- --- --- ... ... ...
Bmglv3 .A. A.A G.A ..C ..C ... .GA G.. .T. ... ... ... --- --- ... ... ..C ..T ..C --- --- --- --- ... ..G ...
Bmglv4 .A. ..A GGA ... ..A ..T .G. G.T ..G ..C ... ... --- --- ... ... ..G ..T ..C --- --- --- --- ... ..C ...

Bmglv1 GAC AAG GAA ATG GGG GGA GGG AAG GTC TTC GGG ACT TTG GGA GAG AGC GAC CAA GGA CTT TTT GGT AAA GGT GGT TAC
Bmglv2 ... ..A C.. ... ... ... ..C ... ... ..T ..C ... ... ... C.A .A. ..T G.T ..C ..C ... ..A ... .C. ... ...
Bmglv3 ... .C. AG. ... ..A ... ... ... ... ... ..C ... ... ... C.. .A. ... G.T ... ..A ..C ... ... .CC ... ...
Bmglv4 ..T ..A C.. G.. ..A ... ... ... ... ... ..C ... C.. ..C C.A .A. ..T G.T ..G ... ... ... ... .CC ... ...

Bmglv1 AAC AGG GAG TTC TTC AAT GAT GAC CGC GGC AAA CTG ACC GGA CAG GCT TAC GGC ACC AGA GTA TTA GGG CCT GGA GGC
Bmglv2 ... .AA ... A.. ..T ... ..C ... ... ... ... ..A ... ..T ... ... ... ..T ... ..G ..T ... ..A ... ... ...
Bmglv3 ... ..A ... A.T ... ... ... ... ... ... ..T C.G ..A ... ..C ... ... ... ... ... ..G ..C ... ..A ..C ... ..T
Bmglv4 ... ..A ... A.. ... ... ... ... ... ... ... T.. ... ..G ... ..C ... ... ... ..G ..C C.G ..A ..C .C. ..G

Bmglv1 GAC AGT ACC AGT TAC GGT GGT CGT CTA GAC TGG GCC AAT GAG AAC GCC AAG GCT GCT ATT GAC TTG AAC AGG CAA ATT
Bmglv2 ... ..C ... .AC ... ..C ..A ..C ... ... ... ..G ..C A.. ..T ..A C.A ..C A.. ... ... C.A ..T ..A ... ..C
Bmglv3 ... ..C ..T .AC ... ... ..A ... ..G ... ... ... ..C A.. ... ..A C.A ... ..C ... ... A.A ... ..A ... ..C
Bmglv4 ... ... ..A .AC ... ... ..A ... ... ... ... ... ... A.. ..T ..G G.A ... ... ... ..T A.A ..T ..A ..G ..C

Bmglv1 GGT GGC AGC GCT GGG ATA GAA GCA TCA GCT TCC GGC GTG TGG GAT CTT GGT AAG AAC ACT CAC TTG TCA GCC GGC GGA
Bmglv2 ... ... ..A T.. ... ..G AC. ... ... .GC ... ..T ... ... ... ..G .A. ... ... ..C ... ..T ..T ... ..T ..T
Bmglv3 ..A ... ..A T.. ... ..G AC. ..C ..G .GC ... ..T ... ... ... ... .A. ... ... ..C ... A.C ... ... ... ..T
Bmglv4 ..A ... ..A T.. ..A ..G AC. ... A.. .G. ..A ..A ... ... ... ... .AC ... ... ..C .G. C.C ... ... ... ..T

Bmglv1 GTG GTC TCT AAG GAG TTC GGT CAC AGA AGG CCT GAT GTC GGT TTA CAG GCC CAG ATT ACT CAC GAG TGG TAA
Bmglv2 A.. ... ..G ... ... ... ... ... .A. ..A ..A ..C ... ... C.T ..A ..A G.. ..C CGC ..T ..T ... .G.
Bmglv3 A.. ... ..G ... ..A ..T ... ... ... ..A ..A ..C ..T ... C.T ..A ..A G.. ..C CGG ..T ... ... .G.
Bmglv4 A.. A.. ..G ... ..A ... ... ... ... ..A ..G ..C ... ..C G.C ... ..A G.. T.C CGC ..T ..T ... .G.
```
