# Supplementary material for: Functional Divergence among Silkworm Antimicrobial Peptide Paralogs by the Activities of Recombinant Proteins and the Induced Expression Profiles
Source: PLoS One. 2011 Mar 29;6(3):e18109. doi: 10.1371/journal.pone.0018109 (PMC3066212; doi:10.1371/journal.pone.0018109)
Supplement: Figure S5 — Sequence alignments of protein sequences for cecropin family (A), moricin family (B) and gloverin family (C). The sequences were aligned using the ClustalX and regions of homology were highlighted using BoxShade (http://www.ch.embnet.org/software/BOX_form.html). (PDF) [file pone.0018109.s005.pdf]

Figure S5

A

```
                    *        20         *        40         *        60
BmcecA1 : MNFVRILSFVFALVLALGAVSAAPEPRWKLFKKIEKVGRNVRDGLIKAGPAIAVIGQAKSLGK-- : 63
BmcecA2 : MNFVRILSFVFALVLALGAVSAAPEPRWKLFKKIEKVGRNVRDGLIKAGPAIAVIGQAKSLGK-- : 63
BmcecB5 : MNFAKILSFVFALVLALSMTSAAPEPRWKIFKKIEKMGRNIRDGIVKAGPAIEVLGSAKAIGK-- : 63
BmcecB6 : MNFAKILSFVFALVLALSMTSAAPEPRWKIFKKIEKMGRNIRDGIVKAGPAIEVLGSAKAIGK-- : 63
BmcecB3 : MNFAKILSFVFALVLALSMTSAAPEPRWKIFKKIEKMGRNIRDGIVKAGPAIEVLGSAKAIGK-- : 63
BmcecB4 : MNFAKILSFVFALVLALSMTSAAPEPRWKIFKKIEKMGRNIRDGIVKAGPAIEVLGSAKAIGK-- : 63
BmcecB1 : MNFAKILSFVFALVLALSMTSAAPEPRWKIFKKIEKMGRNIRDGIVKAGPAIEVLGSAKAIGK-- : 63
BmcecB2 : MNFAKILSFVFALVLALSMTSAAPEPRWKIFKKIEKMGRNIRDGIVKAGPAIEVLGSAKAIGK-- : 63
BmcecC  : MNFVKILCVVLALMLALSMASAALEPKRKVFKIIEKIGRNVRGGVITAGPAVVVVGQAASVGM-- : 63
BmcecE  : MNFSRALFYVFAVFLVCASVMAAPEPRWKIFKKIEKVGQNIRDGIIKAGPAVAVVGQAATIAHGK : 65
BmcecD  : MKISKIFVFVFAIVFATASVSAAPG---NFFKDLEKMGQRVRDAVISAAPAVDTLAKAKALGQG- : 61
          Mnf 4il  VfA6 lal   sAApep wk FKk6EK6Grn6Rdg66kAgPA6 v6g Ak 6g
```

B

```
                    *        20         *        40         *        60
BmmorB7 : MKVFSFFCVVLAMLVLIMGGTSAAPEPKGIGKIIRKGGKVIKHGLTAIGVGAAGHEVYQDSKNSG--- : 65
BmmorB8 : MKVFSFFCVVLAMLVLIMGGTSAAPEPKGIGKIIRKGGKVIKHGLTAIGVGAAGHEVYQDSKNSG--- : 65
BmmorB3 : MKVFSFFCVVLAMLVLIMGGTSAAPEPKGIGKIIRKGGKVIKHGLTAIGVGAAGHEVYQDSKNSG--- : 65
BmmorB1 : MKVFSFFCVVLAMLVLIMGGTSAAPEPKGIGKIIRKGGKVIKHGLTAIGVGAAGHEVYQDSKNSG--- : 65
BmmorB2 : MKVFSLFCVVLAMLVLIMGGTSAAPEPKGIGKIIRKGGKVIKHGLTAIGVGAAGHEVYQDSKNSG--- : 65
BmmorB4 : MKVFSIFCVVLAMLVLIMGGTSAAPEPKGIGKIIRKGGKVIKHGLTVIGVGAAGHDAYQQSQNSG--- : 65
BmmorB5 : MKVFSLFCVVLAMLVLIMGGTSAAPEPKGIGKIIRKGGKIIKHGLTVIGVGAAGHDAYQQSQNSG--- : 65
BmmorB6 : MKVFSIFCVVLAMLVLIMGGTSAAPEPKGIGKIIRKGGKVIKHGLTVIGVGAAGHEVYQESKNSG--- : 65
BmmorA2 : MYFLKYFIVVLVALSLMICSGQADPKIP--VKSLKKGGKIIAKGFKVLTAAGTAHEVYSHVRNRGNQG : 66
BmmorA3 : MYFLKYFIVVLVALSLMICSGQADPKIP--VKSLKKGGKIIAKGFKVLTAAGTAHEVYSHVRNRGNQG : 66
BmmorA1 : MDFLKYFIVVLVALSLMVCSGQADPKIP--VKSLKKGGKIIAKGFKVLTAAGTAHEVYSHVRNRGNQG : 66
Bmmor   : MNILKLFLVFMVAMSLVSCSTAAPAKIP--IKAIKTVGKAVGKGLRAINIASTANDVFNFLKPKKRKH : 66
          M     F Vv6  6 L6     A p     K 64kgGK 6  G   6      h v5    n g
```

C

```
                  *        20         *        40         *        60         *        80         *
Bmglv3 : MNSKLLFFIATVLVCVNAEVYRSSDYEKEYPIRGLFSK--RHPRD----VTWDTRMGGGKVFGTLGQNDDGLFGKAGYNREIFNDDRGQL :  84
Bmglv4 : MNSKLLYFFATVLVCVNAEVY--SEYEEGYPISGQFSK--RHPRD----VTWDKQVGGGKVFGTLGQNDDGLFGKAGYNREIFNDDRGKL :  82
Bmglv2 : MNSNLFYIFATTLVCVNAEVYGPSDYAEDYSISGQSSR--RHPRD----VTWDKQMGGGKVFGTLGQNDDGLFGKAGYNKEIFNDDRGKL :  84
Bmglv1 : MYSKVLLS-AALLVCVNAQVSMPPGYAEKYPITSQFSKSVRHPRDIHDFVTWTREMGGGKVFGTLGESDQGLFGKGGYNREFFNDDRGKL :  89
         MnSk6l    At LVCVNA2Vy   s Y e YpI gqfS4   RHPRD    VTWd  6GGGKVFGTLG2nDdGLFGKaGYN4EiFNDDRGkL

                100         *       120         *       140         *       160         *
Bmglv3 : TGQAYGTRVLGPGGDSTNYGGRLDWANKNAQAAIDINRQIGGRSGMTASGSGVWDLDKNTHISAGGMVSKEFGHRRPDVGLQAEIRHEW : 173
Bmglv4 : TGQAYGTRVLGPAGDSTNYGGRLDWANKNAEAAIDINRQIGGRSGMTATGSGVWDLDKNTRLSAGGMISKEFGHRRPDVGVQAEFRHDW : 171
Bmglv2 : TGQAYGTRVLGPGGDSTNYGGRLDWANKNAQATIDLNRQIGGRSGMTASGSGVWDLDKNTHFSAGGMVSKEFGHKRPDVGLQAEIRHDW : 173
Bmglv1 : TGQAYGTRVLGPGGDSTSYGGRLDWANENAKAAIDLNRQIGGSAGIEASASGVWDLGKNTHLSAGGVVSKEFGHRRPDVGLQAQITHEW : 178
         TGQAYGTRVLGPgGDSTnYGGRLDWANkNA AaID6NRQIGGrsG6tA3gSGVWDLdKNTh SAGG66SKEFGH4RPDVG6QA2irH W
```
