# Supplementary material for: Functional Divergence among Silkworm Antimicrobial Peptide Paralogs by the Activities of Recombinant Proteins and the Induced Expression Profiles
Source: PLoS One. 2011 Mar 29;6(3):e18109. doi: 10.1371/journal.pone.0018109 (PMC3066212; doi:10.1371/journal.pone.0018109)
Supplement: Table S1 — Cloning primers for recombinant expression of antibacterial peptides. (PDF) [file pone.0018109.s007.pdf]

Table S1 Cloning primers for recombinant expression of antibacterial peptides

Name	Forward primer	Reverse primer	Forward primer
BmcecA1	CATGCCATGGTTAGGTGGAAACTCTTCAAG	GATATCTCGAGTCATAAGGATTTCGCTTGCCCTATG	
BmcecB6	CATGCCATGGTTAGGTGGAAGATCTTCAAG	GACCCTCGAGTCAGATAGCTTTAGCCGAACCAAGG	
BmcecC	CGCGCCATGGTTAAACGGAAGGTCTTCAAAATAA	ATATCTCGAGTCAGACGGATGCCGCTTGTCCAACG	
BmcecD	CAGTCCATGGGCAACTTCTTCAAGGATC	CCGCTCGAGTCATTGTCCGAGAGCTTTTGCTTTTG	
BmcecE	CATGCCATGGTTAGATGGAAGATTTTCAAG	ATATCTCGAGTCAGATGGTCGCCGCCTGCC	
Bmglv1	CGAACCATGGTTTCTATGCCTCCTGGTTACGC	GCAAGGCCACTCGAGCCACTCGTGAGTAATCTG	
Bmglv2	CATGCATGGTTTACGGACCTTCTGATTACG	AAACTCGAGCCA(A/C)TCATG(G/C)CGGA(A/T)CTCTG	
Bmglv3	GCGCGCCATGGTATACAGGTCTTCTGATTATG	AAACTCGAGCCA(A/C)TCATG(G/C)CGGA(A/T)CTCTG	
Bmglv4	GTAACCATGGTTTATTCGGAGTACGAAGAAGGATATCCA(A/G)TTAG	AAACTCGAGCCA(A/C)TCATG(G/C)CGGA(A/T)CTCTG	
Bmmor	CATGCCATGGCAAAAATACCTATCAAGGCCATTAAGACTGTAGGAAAGGCAGTCGGTA	CCGCTCGAGTCAATGCTTTCTTTTCTTCGGTTTCAAGAAATTGAAAACATC	GAAAGGCAGTCGGTAAAGGTCTAAGAGCCATCAATATCGCCAGTACAGCCAACGATGT
BmmorLA1	CATGCCATGGCAAAAATTCCTGTCAAAAGTCTGAAGAAGGGTGGAAAAATTA	CCGCTCGAGTCAACGCACGTGGCTATAGACTTCATGGGCTGTTCCCGC	AAGGGTGGAAAAATTATTGCCAAAGGCTTTAAAGTTCTCACCGCAGCGGGAACA
BmmorLB1	CATGCCATGGAACCTAAGGGCATCGGGAAAATCATCAGAAAGGGCGGGAAA	CCGCTCGAGTCATCCGCTGTTTTTCGAGTCTTGGTAGACCTCGTGTCC	CAGAAAGGGCGGGAAAGTTATTAAACATGGACTTACCGCAATAGGCGTAGGAGCTGCCG
BmmorLB5	CATGCCATGGAACCTAAGGGCATCGGGAAAATCATCAGAAAGGGCGGGAAA	CCGCTCGAGTCATCCGCTGTTCTGCGACTGTTGGTAGGCGTCG	CAGAAAGGGCGGGAAAATTATTAAACATGGGCTGACCGTCATCGGCGTCGGGGCCGCCG
BmmorLB6	CATGCCATGGAACCTAAGGGCATCGGGAAAATCATCAGAAAGGGCGGGAAA	CCGCTCGAGTCAGCCGCTGTTCTTTGACTCTTGGTAGACTTCATGTCC	CAGAAAGGGCGGGAAAGTTATTAAACATGGATTAACCGTAATCGGCGTTGGAGCCGCAG
