# Supplementary material for: Functional Divergence among Silkworm Antimicrobial Peptide Paralogs by the Activities of Recombinant Proteins and the Induced Expression Profiles
Source: PLoS One. 2011 Mar 29;6(3):e18109. doi: 10.1371/journal.pone.0018109 (PMC3066212; doi:10.1371/journal.pone.0018109)
Supplement: Table S2 — Primers for quantitive real-time RT-PCR. (PDF) [file pone.0018109.s008.pdf]

Table S2 Primers for quantitive real-time RT-PCR

Name	Forward primer	Reverse primer
BmcecA1	CGTACGTATTTTGAGCTTCGTCTTC	AGGATTTCGCTTGCCCTATGA
BmcecB6	GCAAAGATCCT ATCCTTCGTC	GAACCAAGGACCTCGATCGCC
BmcecD	GTTTTCGCCACGGCTTCGGTCTC	GTCCGAGAGCTTTTGCTTTTGCCAG
BmcecE	CTCAAGAGCTCTGTTTTACGTTTTC	TGTTCTGACCCACCTTTTCG
Bmglv1	AAGGTGTTGTTATCCGCTG	GCAAAGTCGTGAATATCGC
Bmglv2	ATTACTCGATCAGCGGGCAATCC	GCCGTAGTTGGTGCTGTCG
Bmglv3	GCCGCAGAAGTATACAGGTC	CGGAGCCCGAGGCTGTC
Bmglv4	CTATTATATTTCTTCGCCACG	CGGAACTCTGCCTGGACG
Bmmor	GGCAATGTCTCTGGTGTCATGTAG	GCTTTCTTTTCTTCGGTTTC
BmmorLA1	ATTGATGGTTTGCAGTGGACAGGC	ACCTTGATTCCCACGGTTACGCACG
BmmorLB6	GTTCAGTATATTCTGCGTGG	TCTTTGACTCTTGGTAGACTTC
Actin A3	CGGGAAATCGTTCGTGAT	ACGAGGGTTGGAAGAGGG
